# Effect of increased *p*CO_2_ in seawater on survival rate of different developmental stages of the harpacticoid copepod *Tigriopus japonicus*

**DOI:** 10.1080/19768354.2017.1326981

**Published:** 2017-05-22

**Authors:** Je Hyeok Oh, Dongsung Kim, Tae Won Kim, Teawook Kang, Ok Hwan Yu, Wonchoel Lee

**Affiliations:** a Marine Ecosystem and Biological Research Center, Korea Institute of Ocean Science and Technology, Ansan, Republic of Korea; b Department of Life Science, Hanyang University, Seoul, Republic of Korea

**Keywords:** Ocean acidification, carbon dioxide, harpacticoid copepod, *Tigriopus japonicus*, survival rate

## Abstract

The rapid increase in carbon dioxide levels in seawater is causing ocean acidification and is expected to have significant effects on marine life. To explore the ability of the harpacticoid copepod *Tigriopus japonicus* to adapt to an increased concentration of dissolved carbon dioxide (CO_2_) in seawater, we compared the survival rates of adult and nauplius stages at 400, 1000, and 1550 ppm *p*CO_2_ over a 14-day period. The survival rate of *T. japonicus* dramatically decreased over time with increase in *p*CO_2_ concentration. At 1550 ppm, the survival rate showed a decrease of more than 20% at the end of the experimental period over that at 400 ppm. Furthermore, the survival rate decreased by a greater amount at all concentrations in nauplii than in adults, with a greater effect in wild-collected specimens than in culture-derived individuals. The results suggest that future ocean acidification may negatively influence the sustainability of *T. japonicus* and thus may eventually influence benthic ecosystems.

## Introduction

In the 200 years since the industrial revolution, the concentration of carbon dioxide (CO_2_) in the atmosphere has increased from 280 to 380 ppm due to human activity (Feely et al. 2004). The recent climate change report by the IPCC predicted that, at the current rate of increase in CO_2_ concentration in the atmosphere, the amount of CO_2_ in the ocean will increase to 851–1370 ppm by 2100, and to 1371–2900 ppm by 2150 (IPCC 2014).

The increase in the concentration of CO_2_ dissolved in the ocean will induce carbonate ion deficiency, which will reduce the productivity and survival of marine organisms that have calcium carbonate shells and frames (Gattuso et al. 1998; Riebesell et al. 2000) and also have an adverse effect on physiological functions such as development (Fabry et al. 2008), metabolism (Pörtner 2008), ion adjustment (Pörtner et al. 2004), and acid–base equilibrium (Widdicombe & Spicer 2008).

A number of studies have examined the effects of ocean acidification on marine life. However, most studies on benthic invertebrates have focused on macrobenthic organisms such as abalone (Kim et al. 2013), clams (Lee & Kim 2016), mussels, hermit crabs (Kim et al. 2016; Kim & Barry 2016), and sea urchins (Sung et al. 2014). Smaller benthic organisms such as the meiofauna are less well studied (Dupont & Thorndyke 2009; Przeslawski et al. 2009). Additionally, many of the studies on meiofauna addressed the effect of high concentrations of CO_2_ (>5000 ppm) in relation to CO_2_ capture and storage projects (Barry et al. 2005; Thistle et al. 2006; Pascal et al. 2010; Kita et al. 2013); there have been no studies on the effects of long-term exposure to relatively low CO_2_ concentrations (<1600 ppm).

Harpacticoid copepods form one of the dominant taxa in the meiobenthos along with nematodes, and are present in a range of habitats from the deep-ocean to coastal areas. They have a very important role in the food chain of coastal ecosystems. Harpacticoid copepods can be used as indicator species because they are very sensitive to environmental changes and pollution, and thus serve as an important tool for environmental assessment (Ito 1970; Kusk & Wollenberger 1999). The responses of harpacticoid copepods to environmental changes differ among life cycle stages; thus, both adult and nauplius stages need to be examined in experimental studies (Forget et al. 1998). *Tigriopus japonicus* is a harpacticoid copepod species that inhabits the coastal intertidal zone; it is present in large numbers at a site and can easily be cultured in the laboratory. As it is sensitive to environmental changes, *T. japonicus* is often used as an experimental organism (Lee 1991; Barka et al. 2001; Cao et al. 2015).

The purpose of the present study was to determine the effects of ocean acidification on *T. japonicus* and examine whether these effects differ among growth stages and environments. We determined changes in survival rates in adults and nauplii of *T. japonicas* which have acclimated to the different growth environments after exposure to increased seawater CO_2_ concentrations.

## Materials and methods

### Test organism

The experiments were carried out using two groups of *T. japonicus*, namely laboratory cultured copepods and wild-caught copepods. The former were provided by the copepod culture laboratory of Gangneung-Wonju National University, Republic of Korea in May 2012, and maintained in an incubator at a constant temperature (20°C) for one week. Seawater was used as the culture medium after filtration through 1.2 µm pore size GF/C filter paper. The photoperiod was adjusted to 12 h light:12 h dark and the salinity of the seawater was maintained at 28.0–30.0 psu. The *T. japonicus* were fed a mixture of green algae (*Tetraselmis suecica*) and haptophyta (*Isochrysis galbana*) in a ratio of 1:1; each copepod was supplied with 3 µL of the mixture once per day during the culture period. The wild-caught copepods were obtained from an intertidal zone on the eastern coast of Hupo harbor in Uljin in May 2012; they were acclimatized to a salinity concentration of 30.0 psu and temperature of 20°C for one day.

At the end of the experiment, wild-caught organisms were fixed in 70% ethanol; their identity was confirmed by dissection and analysis under a dissection microscope (Leica MZ16) and optical microscope (Olympus BX51).

### CO_2_ supply and *p*CO_2_ control

**Figure 1.**
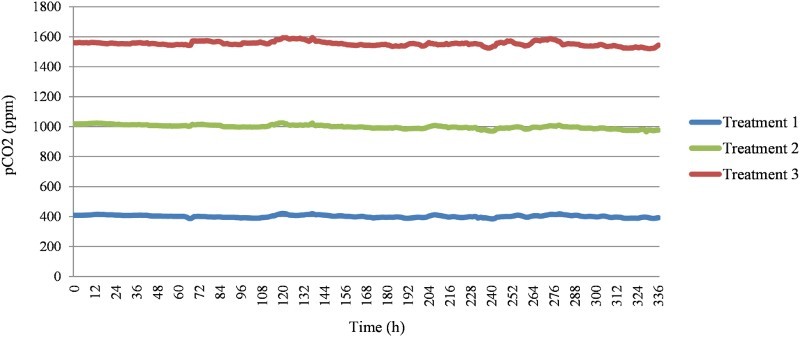
Values of *p*CO_2_ monitored in each treatment during the experimental period of 14 days.

For the experiment, seawater at a constant *p*CO_2_ concentration was supplied using the AICAL system, which is a high-accuracy CO_2_ automatic manipulation system (Fujita et al. 2011; Suwa et al. 2013). This system includes CO_2_ dissolution and measurement towers, which facilitate the effective dissolution of gaseous CO_2_ into seawater and the continuous logging of *p*CO_2_ levels and water temperature in the generated seawater. The desired levels of *p*CO_2_ gas were generated by blending pure CO_2_ gas with CO_2_-reduced air in a *p*CO_2_-regulation system (CGM-07 and DGG-07; Kimoto Electric, Osaka, Japan), which is part of the AICAL system. The *p*CO_2_ levels were logged every hour using a *p*CO_2_ control/monitoring system (CO2-07; Kimoto Electric). A range of *p*CO_2_ concentration was set from 400 ppm, which is the current average CO_2_ concentration in the atmosphere, to 1550 ppm, which is one of five predicted CO_2_ concentration values by the year 2100 (IPCC 2007). Three CO_2_ treatments were set: 400, 1000, and 1550 ppm. The *p*CO_2_ was monitored each hour during the experimental period. The average *p*CO_2_ concentration was 401.2 ± 7.9 (standard deviation) ppm for treatment 1 (control group), 999.3 ± 12.7 ppm for treatment 2, and 1553.7 ± 15.0 ppm for treatment 3 (Table 1, Figure 1).

**Table 1.** *p*CO_2_, pH, salinity, and temperature measured in each treatment during the experiment.

	*p*CO_2_ (ppm)		pH	Salinity (psu)	Temperature (°C)
	Nominal	Actual			
Treatment 1 (Control)	400	401.2 ± 7.9	8.05 ± 0.01	30.0 ± 0.1	20.0 ± 0.1
Treatment 2	1000	999.3 ± 12.7	7.79 ± 0.01		
Treatment 3	1550	1553.7 ± 15.0	7.61 ± 0.02		

### Experimental chamber

**Figure 2.**
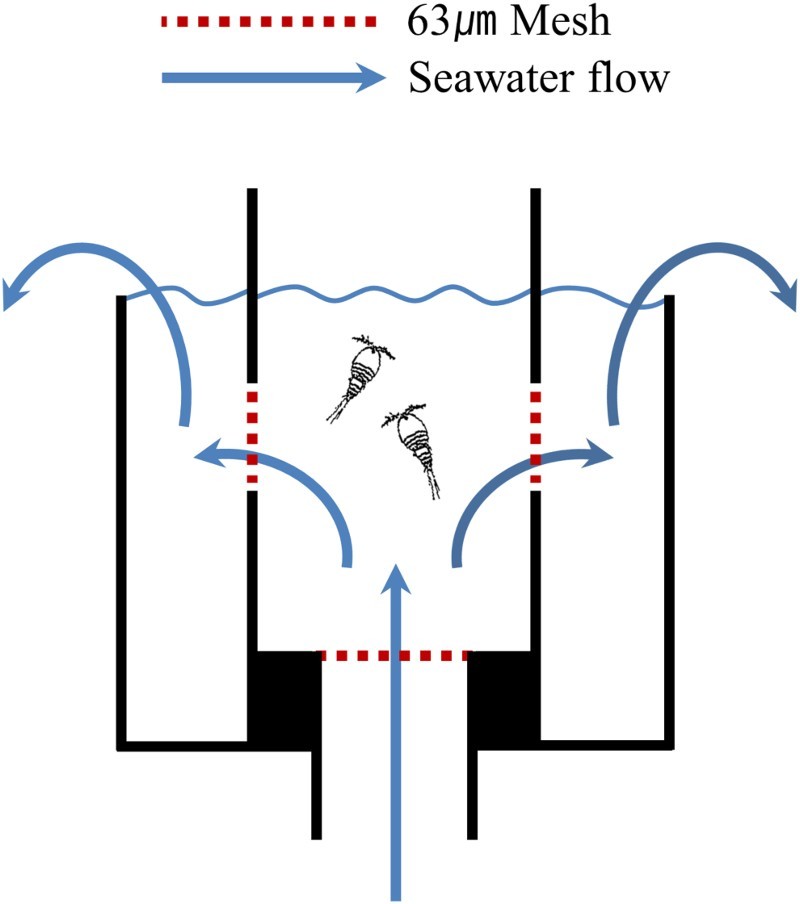
Experimental chamber set.

*T. japonicus* was exposed to the seawater of each experimental treatment in an experimental chamber. The chamber had an open top, a height of 4 cm, and a cylindrical diameter of 2.5 cm; the open top allowed quick observation of surviving and dead individuals through a dissection microscope. The chamber was constructed of transparent acrylic plastic and had a volume of 10 ml. A hole (13 mm diameter) was drilled into the bottom of the chamber, and two holes (10 mm diameter) were made in the sides of the chamber (Figure 2). Each hole was covered by a protective mesh screen (pore size 63 µm) to ensure that the experimental organisms remained within the chamber. Each chamber was linked by a hose to the AICAL system and sweater was pumped continuously through the system at a constant rate of 300 ml/min. The seawater entered the chamber through the hole in the bottom and exited through the two side holes. Three chambers were prepared for each experimental treatment.

### Survival rate measurements

The survival of adults and nauplii at each *p*CO_2_ concentration was examined under the same salinity, temperature, and photoperiod conditions as in the cultures. For the adult group, food was supplied in the same manner as in the cultures. Surviving nauplii grew throughout the experimental period; thus, the amount of food supplied differed at different growth stages, with 0.5 μL supplied to nauplii, 1 μL to copepodites, and 3 μL to adults.

To accurately identify complete adult individuals, brooding female were used. On the other hand, to obtain nauplius stage individuals, 30 brooding females were placed into a 6-well culture plate and stage 1 nauplii were collected and used. Five nauplii were placed in each chamber, and the experiment was carried out for 14 days. To reduce experimental errors, hatching nauplii from brooding females were removed from the adult chamber every 12 h. Measurements were made every 24 h for each concentration, counting dead and surviving individuals and converting these numbers to survival rates.

### Statistical analysis

Significant differences in survival rates among the treatment groups with different *p*CO_2_ concentrations were tested using repeated-measures ANOVA with the SPSS program (IBM SPSS statistics 19.0, SPSS Inc., IBM company). If there was a significant difference in the repeated-measures ANOVA test, Tukey’s HSD (honestly significant differences) test was performed as a *post-hoc* test for the multiple comparison analysis.

## Results

**Figure 3.**
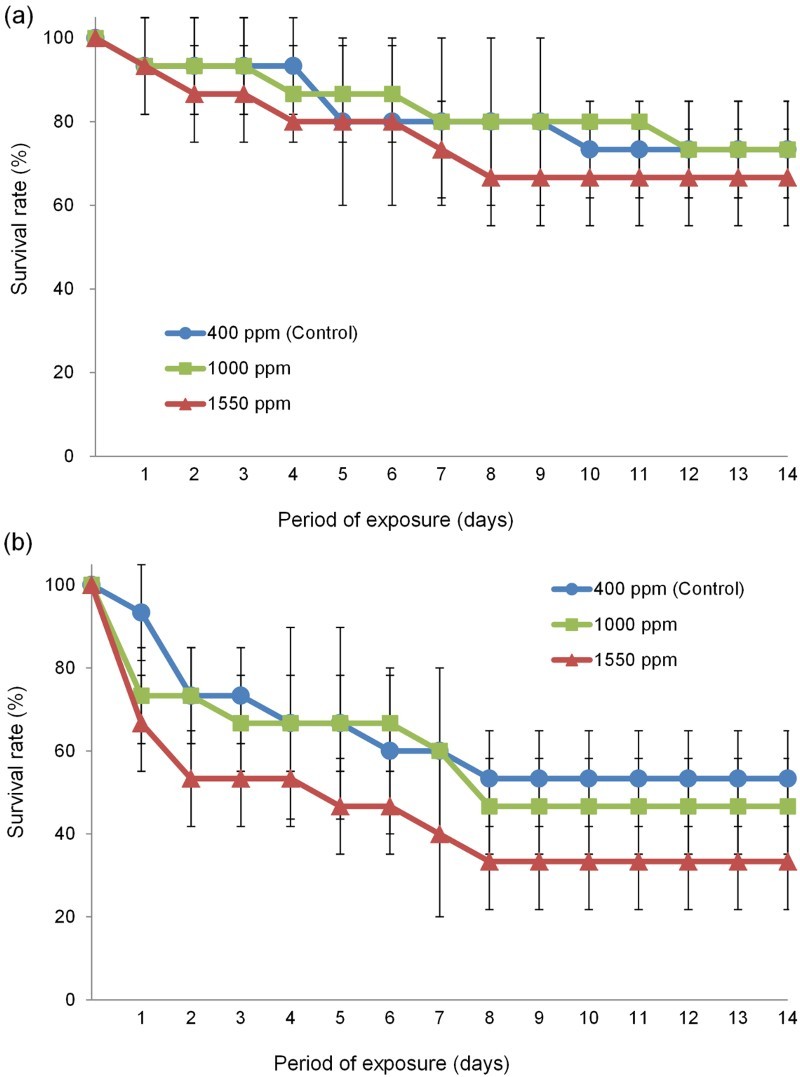
Changes in the survival rates of adult (a) and nauplius (b) of the culture-derived *T. japonicus* exposed to seawater with different *p*CO_2_ for 14 days.

The survival rate did not differ significantly among treatments for adults of the culture groups (*F* = 0.86, d*f* = 2, *P* > .05, Figure 3(a)). However, in the nauplius group, the survival rate at 400 ppm (control) was significantly higher than that at 1550 ppm (*F* = 4.568, d*f* = 1, *P* < .05, Tukey *post hoc* test, *P* < .05, Figure 3(b)).

**Figure 4.**
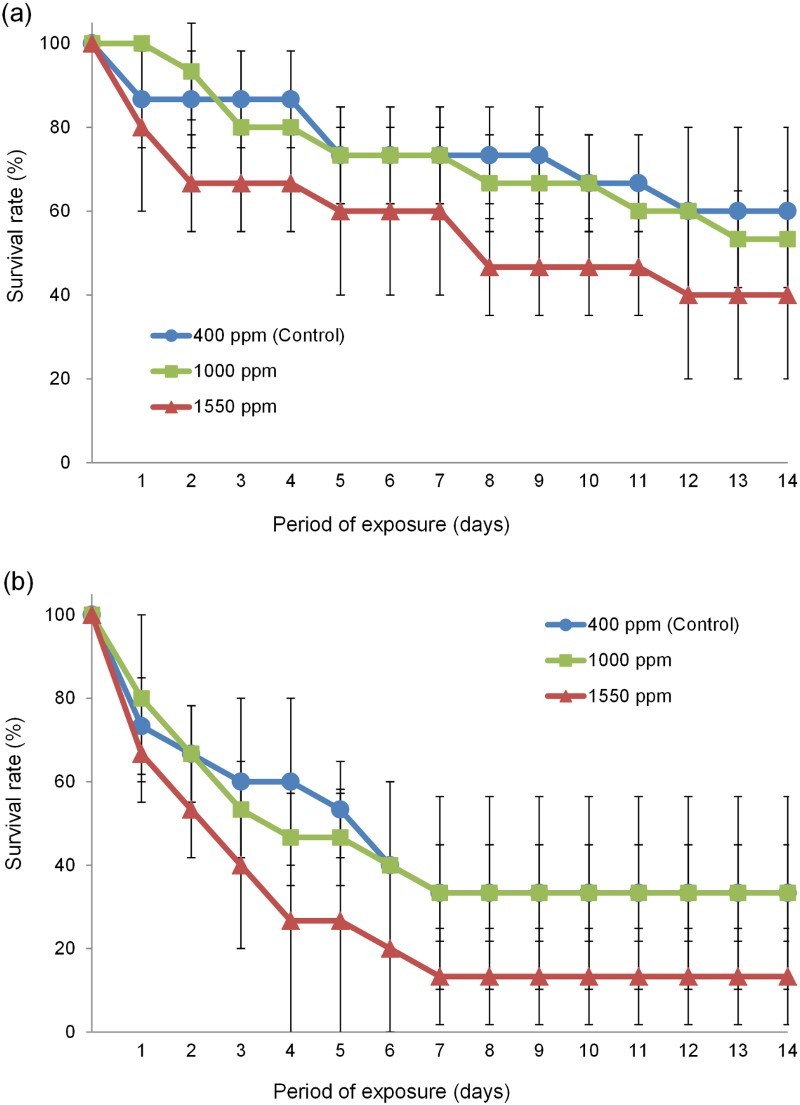
Changes in the survival rates of adult (a) and nauplius (b) of the collected *T. japonicas* exposed to seawater with different *p*CO_2_ during 14 days.

Significant variation in survival rates was found among wild-caught *T. japonicus* after 14 days exposure to different *p*CO_2_ levels (Figure 4). Survival rates of both adults and nauplii were significantly lower at 1550 ppm than in the control (adult: *F* = 4.568, d*f* = 1, *P* < .05, Figure 4(a); nauplius: *F* = 7.636, d*f* = 1, *P* < .05, Figure 4(b)).

## Discussion

The survival rate of *T. japonicus* was significantly lower at the highest *p*CO_2_ concentration tested here, indicating that *p*CO_2_ concentrations negatively affected the organisms. This result suggests that drastic environmental changes such as ocean acidification induce distinct changes to physiological functions in a harpacticoid copepod species that plays a role as a major secondary producer in coastal benthic ecosystems. Marine organisms may use the acid–base equilibrium for acidity control in the body, or limit reproduction and growth for long-term preservation of species (Langenbuch & Pörtner 2004). It has been reported that egg production in copepods is reduced when the organisms are exposed to CO_2_-induced acidified seawater over multiple generations (Mayor et al. 2007; Weydmann et al. 2012; Hildebrandt et al. 2014). The calanoid copepod *Acartia tonsa* showed delayed growth when exposed to a *p*CO_2_ of 1000 µatm that resulted in pH 7.8 (Cripps et al. 2014). In addition, when deep-sea sites are temporarily acidified by exposure to high CO_2_ concentrations, different species of harpacticoid copepod vary in their tolerance to the change (Barry et al. 2005; Thistle et al. 2006). Although there have been many studies on the effects of ocean acidification on copepods, particularly benthic harpacticoid copepods, in different regions in recent years, the amount of information available on their responses is still insufficient and the effects are not clearly defined.

This study showed that there was a significant difference in the survival rates of adult and nauplius *T. japonicus* exposed to 400 ppm from that in *T. japonicus* 1550 ppm (*P* < .05). Our results suggest that survival of *T. japonicus* is reduced at a CO_2_ concentration of 1550 ppm or more (less than pH 7.6). The mechanisms that underlie this difference in survival rate are currently unknown.

In this study, the survival rates of *T. japonicus* nauplii showed greater changes with CO_2_ concentration than did those of adult stages. The nauplius survival rate at 1550 ppm decreased dramatically to half that of the control in the first 2 days of the experiment in both culture-derived and wild-caught groups. The harpacticoid copepod species *T. japonicus* is widely used in toxicity assessment experiments (McAllen et al. 1999; Barka et al. 2001; McAllen & Taylor 2001). These copepods undergo several molts and one metamorphosis during the transformation into an adult after hatching. During growth, the morphological, physiological, and behavioral characteristics between each growth stage show major differences. Accordingly, experiments that include a range of growth stages are considered essential in analyses of the effects of environmental changes of pollutants (Pounds et al. 2002; Fitzer et al. 2012; Cao et al. 2015). Our findings here are consistent with those of previous studies. Nauplii of the harpacticoid copepod *Amphiascus tenuiremis* were shown to be 28 times more sensitive in terms of survival rate than adult individuals to exposure to the organophosphate pesticide chlorpyrifos (Green et al. 1996), while males of the copepod *A. tonsa* had a two-fold higher rate of reaction to toxicity than females (Barata et al. 2002). Moreover, early development stages showed more sensitivity to pollutants (Green et al. 1996; Lotufo 1997). Copepods at early developmental stages showed more sensitivity to CO_2_ storage in deep-sea sites (Pörtner & Farrell 2008), and invertebrates were especially affected at early development stages (Mayor et al. 2007; Dupont & Thorndyke 2009). In the current work, culture-derived and wild-caught nauplii showed a drastic decrease in survival rate for 8 days after the start of the experiment, whereas after 8 days does not decrease any more. This is considered to showing that only individuals with strong tolerance survived except for those which are vulnerable to the effects of acidification.

Our finding that wild-caught adults were more sensitive to increased CO_2_ than culture-derived adults suggests that the latter group is more acclimated to lower pH environments. The pH of the seawater from which the wild-caught groups were obtained was 7.93, whereas that of the cultured group was 7.76. If latter is already adapted to a lower pH environment, it is possible that they might be less sensitive to a decrease in pH. But, contrary to the results of this study, some other studies investigating adult harpacticoids, including *T. japonicus*, found strong resistance and tolerance even at very high concentrations of CO_2_ (Kurihara et al. 2007; Cao et al. 2015). However, referring studies clearly differed from ours in the target individuals used. It was not a taxonomical difference, but the difference between individuals collected at sea and cultivated for several generations in a laboratory. Many of the studies on harpacticoid copepods have used individuals derived from laboratory cultures maintained for many generations (McAllen & Taylor 2001; Fitzer et al. 2012; Kita et al. 2013). Indeed, the aforementioned studies that found a strong tolerance in *T. japonicus* to ocean acidification used culture-derived individuals. However, it is possible that these may have developed physiological differences to wild-living individuals. The results of the current study partially support this conclusion.

Our study of *T. japonicus* found a difference in CO_2_ tolerance between adults and nauplii, as well as between those from the wild and from culture. Our results confirm that *T. japonicus* is a useful experimental organism for investigating ocean acidification. The data showed a decrease in survival with increasing CO_2_ concentration, and this effect was greater in wild-collected individuals than in the culture-derived individuals. We suggest that copepods collected directly from the sea may be more suitable for ocean acidification experiments in consideration of the possibility of the field application and usefulness of the experimental results. Further work is required to determine the extent and nature of physiological changes between wild-living individuals and those in culture. Additionally, the effects of ocean acidification on a range of harpacticoid copepod species needs to be examined and further research into environmental factors that can act as co-stressors is needed to predict the shifts in harpacticoid copepod communities that may be induced in the future.
